# Possible pathogenic mechanism of gluteal pain in lumbar disc hernia

**DOI:** 10.1186/s12891-018-2147-y

**Published:** 2018-07-11

**Authors:** Yu Wang, Jin Yang, Yuqing Yan, Lifeng Zhang, Chuan Guo, Zhiyu Peng, Qingquan Kong

**Affiliations:** Department of Orthopedic Surgery, West China Hospital, Sichuan University, No. 37, Guoxue Lane, Wainan Street, Wuhou District, Chengdu, 610041 Sichuan China

**Keywords:** Gluteal pain, Lumbar disc hernia, Dorsal rami

## Abstract

Recent reported results by Fang et al. published in BMC Musculoskeletal Disorders have added to the weight of evidence supporting association between gluteal pain and lumbar disc hernia. Their clinical finding shows the L4/5 level is the main level responsible for gluteal pain in lumbar disc hernia. Indeed, many possible mechanisms may explain why patients experience pain in the gluteal area. In this Correspondence, we would like to highlight several possible mechanisms of LDH-related gluteal pain based on detailed analysis of the sensory innervation of the gluteal region. We hope this can better explain the phenomenon found by Fang et al. We believe the principle mechanism is compression/irritation of L5 or S1 dorsal rami (intraspinal portion), which produce gluteal pain by irritating superior/medial cluneal nerve and referred pain from facet joints and sacroiliac joints. In addition, the presence of proximal sciatica could also induce gluteal pain. Lastly, fibers in the superior or inferior gluteal nerve could be compressed/irritated in LDH, inducing LDH-related gluteal pain. However, additional studies are needed in the future to delineate the exact mechanism(s).

We read the article “Which level is responsible for gluteal pain in lumbar disc hernia?” [1] published in BMC Musculoskeletal Disorders with great interest; first, we congratulate the authors for their informative work. In the study, the author collected single-level Lumbar Disc Hernia (LDH) patients who underwent Percutaneous Endoscopic Lumbar Discectomy (PELD). All patients were divided into two groups based on the presence of gluteal pain: gluteal pain (GP) and non-GP group. After analyzing the distribution of the levels and surgical outcome between the two groups, the authors confirmed the strong association between LDH and gluteal pain and concluded that L4/5 disc hernia was regarded as the primary factor of LDH-related gluteal pain. We are interested in this topic because we encountered 20 similar LDH-related gluteal pain patients in our department from January 2016 to June 2017.

In the study, a total of 94.6% of the 159 GP patients were L4/5 LDH, a much higher proportion than for L5/S1 (5.4%) and L3/4 (0%), thus indicating the apparent tendency for incidence of LDH-related gluteal pain in L4/5. The gluteal pain disappeared after surgery and reappeared with LDH recurrence, seemingly confirming the hypothesis. However, the author did not analyze the exact mechanism primarily based on the sensory innervation of the buttock region or the reasons for the high incidence in L4/5 LDH. Meanwhile, we cannot explain those patients whose gluteal pain syndrome was not relieved after PELD by this hypothesis. To be better understand, we’d like to talk about some possible pathogenic mechanism of LDH-related gluteal pain based on neuroanatomy of the buttock region.

As we know, sensory innervation of the buttock is complicated and controversial. We should be aware of the fact that the ventral roots include motor neurons, and the dorsal roots include sensory fibers that carry information from receptors in the peripheral nerves [2]. All spinal nerves combined with ventral and dorsal roots contain both motor and sensory fibers; they are designated mixed nerves [3, 4]. Lateral branches of lumbar dorsal rami compose the superior cluneal nerve (L1–3, sometimes L4, L5) and the medial cluneal nerve (S1–3), which innervate the upper-lateral and medial surface of the buttock [5–8]. The superior gluteal nerve (L4-S1) and the inferior gluteal nerve (L4-S2) coming from lumbar ventral rami dominate the movement of gluteus Minimus/Medius/Maximus also conduct their sensation [9–11]. Irritation of any above-mentioned nerves can produce gluteal pain. Other sources of gluteal pain include referred pain from lumbar facet joint [5, 12] or sacroiliac joint (SIJ) [13, 14], proximal sciatica after conservative management of radiculopathy [3], deep gluteal syndrome [15, 16] and dorsal rami injury during PELD, etc. In LDH-related gluteal pain, we believe the principal mechanism is the compression/irritation of the dorsal rami, especially the unseparated nerve fibers in the intraspinal portion, caused by LDH. On the one hand, in addition to L1–3, the lateral branch of L5 dorsal rami frequently join in the superior cluneal nerve. Meanwhile, lateral branches of the S1 dorsal rami form the medial cluneal nerve. Both cutaneous nerves innervate most of the buttock surface. On the other hand, facet joints innervated by each medial branch of dorsal rami and SIJ innervated by L4-S1 dorsal rami dorsally produce referred pain in buttock region when L5 and S1 dorsal rami, e.g., L4/5 or L5/S1 LDH, are compressed and irritated. In addition, as others reported, the presence of proximal sciatica could induce gluteal pain when L4-S1 nerve roots are injured [3]. Lastly, L5 and S1 ventral rami are also involved in the superior and inferior gluteal nerves that mediate sensation in the three major muscles of the buttock. Therefore, any possible mechanism mentioned above may produce gluteal pain in LDH, especially in L4/5 or L5/S1 level, which affect L5 and S1 nerve roots, respectively. More favorable studies about pathogenic mechanism of gluteal pain are needed in the future.

## Abbreviation

GP: Gluteal pain
LDH: Lumbar disc hernia
PELD: Percutaneous Endoscopic Lumbar Discectomy
SIJ: Sacroiliac joint
